# Brazil’s research budget: endless setbacks

**DOI:** 10.17179/excli2020-2887

**Published:** 2020-09-21

**Authors:** Lucindo José Quintans-Júnior, George Rego Albuquerque, Sérgio Campello Oliveira, Robério Rodrigues Silva

**Affiliations:** 1 Universidade Federal de Sergipe (UFS). São Cristóvão, Sergipe, CEP 49100-000, Brazil; 2 Universidade Estadual de Santa Cruz (UESC). Ilhéus, Bahia, CEP 45662-900, Brazil; 3 Universidade de Pernambuco (UPE). Recife, Pernambuco, CEP 50100-010, Brazil; 4 Universidade Estadual do Sudoeste da Bahia (UESB). Vitória da Conquista, Bahia, CEP 45083-900, Brazil

## ⁯⁯⁯

***Dear Editor, ***

The budget cuts in science and technology areas in Brazil are making the situation untenable for researchers. There is a lack of money to maintain even basic services, and labs are shutting down. The proposed 2021 draft budget would result in a 29 % reduction in science funding since 2015. If this funding cut is not reversed, it will severely damage the current system of scholarships and research. These endless cuts in science funding need to be reviewed by Brazilian Government and the National Congress, or Brazilian science will be doomed to continued destruction and dismay for researchers.

The crisis in education in Brazil is not a crisis; it is a project', this well-known phrase by the renowned Brazilian anthropologist and writer, Darcy Ribeiro (1922-1997) (Santos and Sampaio, 2017[4]), now also holds true for the area of science. Although there have been cuts in funding under previous governments, the cuts proposed by the current Bolsonaro government are based on an anti-science ideology. The panaceas and miracle cures proposed by the current government for COVID-19 have been yet another embarrassing episode, throwing science into the gutter, and illustrating the current prevalence of a pandemic of ignorance and anti-science attitudes - further exemplified by the disrespect shown to doctors, frontline health workers and family of patients who have died because of the COVID-19 outbreak (Quintans-Júnior and Silva, 2020[3]). 

**Figure 1:**
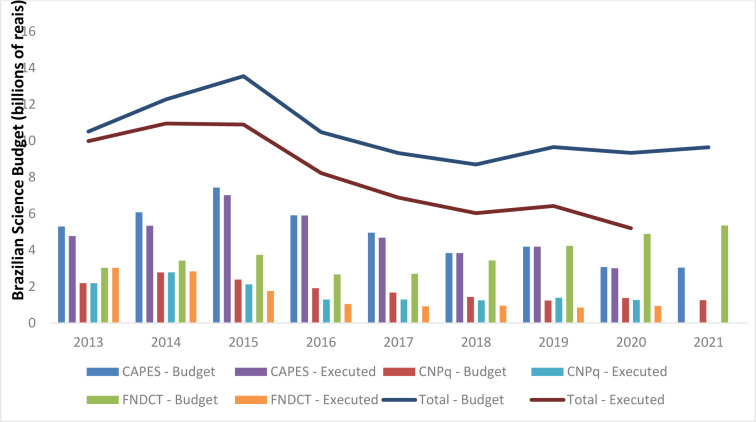
The gap between Brazilian science budget and funding really spent (executed) since 2013

Recently, the Brazilian government presented its draft 2021 budget bill (PLOA) to congress, which includes proposals for science funding for 2021. Despite the science budget on the face of it having a small increase when compared to 2020, in respect of the two main agencies responsible for university and higher education funding in Brazil, CAPES and CNPq, there is a proposed reduction of almost 4 %. A closer analysis of the data reveals a 29 % budget reduction for universities and higher education between 2015 and 2021 (R$ 13.5 billion to R $ 9.6 billion) (PLOA, 2021[2]; Angelo, 2017[1]) and an unacceptable 41 % cut in research funding from 2015 to 2019. Figure 1(Fig. 1) shows a clear gap between forecast budgets and what was actually spent on funding Brazilian science, all against a background of the demagogic rhetoric of populism of the current government (2020 consider the 2015-2019 average execution of forecasting). One of the most concerning points illustrated by the figure is in relation to FNDCT funding (PLOA, 2021[2]; Angelo, 2017[1]). This was a fund created to finance Brazilian science and innovation, but the data clearly shows any increasing gap between the funds theoretically available (budget), and those actually spent (executed) on projects for the development of science in Brazil. These data reinforce the idea that the FNDCT was a project designed solely to disrupt current funding structures and discourage research workers. The gap between the budget and actual spending is on track to be wider than ever in 2020. 

The situation is made even worse by the depreciation of the Brazilian currency, the Real, against the US Dollar, with its value falling almost 60 % from 2015 to 2020 (Trading Economics, 2020[6]), so this makes things worse because a lot of research depends on equipment and materials bought from abroad (particularly the USA and Europe). The situation in Brazil has become so desperate that scientists, as physicist Luiz Davidovich, has said 'an atomic bomb strike on Brazilian science' (Angelo, 2017[1]).

The COVID-19 outbreak should have emphasized the importance of science and technology in the search to understand the disease, and develop new treatments and vaccines, but in Brazil, the atmosphere of scientific denial defended by Bolsonaro and his supporters has created a surreal situation, and a complete lack of strategic vision: Brazil has been without a Minister of Health for the last 110 days, having an interim minister (an army general with no medical experience) during the worst pandemic in the last 100 years. The 2021 draft budget also contains an inexplicable (given that fact that Brazil is in the middle of a major health crisis) proposed reduction of around 22 % (PLOA, 2021[2]) to the health budget, cuts that will not only undermine essential health services, but also mean that important clinical research will have to be stopped.

The dramatic reduction in funding over recent years has already produced deleterious effects, with the number of research papers published declining, reflecting the fact that laboratories are reducing their activities. The number of doctors being trained, and the numbers of Master's students are also falling, and a brain drain is accelerating because leading researchers are finding it impossible to continue their work in Brazil (Scalzaretto, 2020[5]). 

The Brazilian congress should urgently reconsider these proposed budget cuts, review this destructive policy, and impose conditions on the government that ensure that science and higher education in Brazil receive appropriate funding, if they do not, a scorched earth scenario will be seen in a short time in relation to science in Brazil.

## Conflict of interest

The authors declare no conflict of interest.
